# Distribution of Microbial Keratitis After Penetrating Keratoplasty According to Early and Late Postoperative Periods

**DOI:** 10.4274/tjo.galenos.2020.77026

**Published:** 2020-08-26

**Authors:** Onur Özalp, Eray Atalay, Zülfiye Köktaş, Nilgün Yıldırım

**Affiliations:** 1 Eskişehir Osmangazi University Faculty of Medicine, Department of Ophthalmology, Eskişehir, Turkey; 2 Burdur Gölhisar State Hospital, Clinic of Ophthalmology, Burdur, Turkey

**Keywords:** Microbial keratitis, penetrating keratoplasty, keratitis

## Abstract

**Objectives:**

The aim of this study was to investigate the distribution of microbial agents in the early and late postoperative periods in patients with microbial keratitis (MK) after penetrating keratoplasty (PK).

**Materials and Methods:**

The records of 36 patients who were clinically diagnosed as having MK after PK were retrospectively reviewed. Culture results were obtained from microbiology records and the organisms that were produced were noted. A case was deemed as viral keratitis based on the clinical appearance, negative cultures, and response to antiviral treatment. Keratitis development times were evaluated in 2 categories: early (within the first year) and late (after year 1) postoperative period. Mann-Whitney U and Kruskal-Wallis tests were used to compare numerical variables that did not show normal distribution and chi-square test was used to compare categorical variables.

**Results:**

The majority of MK cases were of bacterial origin (55.5%, n=20), followed by viral (41.7%, n=15) and fungal (2.8%, n=1). Of the 15 cases of early postoperative MK, 10 were bacterial, 4 were viral, and 1 was fungal; however, among cases of late postoperative MK, 10 were bacterial and 11 were viral. The majority (65%) of early and late bacterial infections were caused by gram-positive strains (most commonly staphylococci). Gram-positive bacteria caused keratitis significantly earlier than gram-negative bacteria (p=0.037). Viral and gram-negative bacterial MK was more frequent in the late postoperative period, but the difference was not statistically significant.

**Conclusion:**

In our study, bacterial keratitis was more common in post-keratoplasty MK than viral and fungal keratitis. Gram-positive bacteria were the most common causative agents. The increased incidence of gram-negative bacterial agents and viral keratitis in the late postoperative period can be explained by long-term topical steroid use.

## Introduction

In patients who undergo penetrating keratoplasty (PK), microbial keratitis (MK) can occur at any time after surgery and cause severe ocular morbidities such as graft failure and visual impairment.^1,2,3^ The prevalence of MK after PK has been reported as between 1.8% and 12.1%.^1,2,4,5,6^ Loose sutures, retained sutures, and topical steroid use are among the risk factors for MK in eyes with keratoplasty.^7,8^ Early diagnosis and prompt treatment are important to reduce ocular morbidity.^9^

A variety of factors may play a role in the development of MK after penetrating keratoplasty.^2,8,10^ Gram-positive bacteria in the ocular surface flora, most frequently Staphylococcus species, have been identified as the most common causative agents in MK after PK.^3,6^ Gram-negative and fungal keratitis are less common than those caused by gram-positive bacteria.^10^

Herpes simplex virus (HSV) keratitis manifests clinically as classical dendritic or geographic ulcer, or as epithelial defect in atypical forms that are refractory to standard treatment.^11,12,13,14^ The high rate of graft failure associated with HSV keratitis makes early diagnosis and treatment important.^14^

MK can occur in the early or late postoperative period after PK.^10^ Early MK is usually due to ocular surface problems in the recipient, contamination of the donor cornea, and intraoperative contamination, whereas later MK is believed to be due to environmental pathogens.^9,15,16,17,18^

The aim of this study was to investigate the causative agents of MK and analyze their distribution in the early and late postoperative periods after PK in patients whose primary indication for keratoplasty was not keratitis and who did not undergo therapeutic keratoplasty due to infection.

## Materials and Methods

Patients who underwent PK for indications other than MK between 2002 and 2019 in the ophthalmology department of Eskişehir Osmangazi University (ESOGU) Faculty of Medicine were retrospectively evaluated and 36 eyes of 36 patients with post-PK MK were included in the study. Exclusion criteria were pre-PK history of bacterial, viral, or fungal keratitis and undergoing therapeutic keratoplasty for this reason. The study protocol was planned in accordance with the Declaration of Helsinki and approved by the ESOGU Ethics Committee. The patients’ age, sex, systemic diseases, use of antiglaucomatous medication, keratoplasty indications, postoperative time of MK, and corneal scraping smear, culture, and antibiogram results were noted.

Presence of bacterial or fungal keratitis was confirmed by positive direct smear and cultures of corneal scraping sample; viral keratitis was diagnosed in patients who responded to antiviral treatment initiated based on clinical presentation and had negative culture.

Polymicrobial keratitis was defined as an infection in which multiple pathogens were identified, regardless of whether they were bacterial, viral, or fungal.^10^ Time of keratitis development was classified as in the early (within the first year) or late (after 1 year) postoperative period.

Blepharitis or nasolacrimal duct obstruction was not detected in any of the patients in ophthalmologic examination before keratoplasty. All patients who underwent keratoplasty received postoperative topical antibiotics for at least 4 to 6 weeks and topical steroid therapy tapered over a period of at least 1 year. Topical antibiotic treatment was initiated empirically (e.g., combined vancomycin-amikacin) in patients who developed MK and was adjusted according to smear and culture results.

### Statistical Analysis

All statistical analyses were performed using IBM SPSS Statistics version 21.0 (IBM Corp., Armonk, NY). Normal distribution of the variables was tested using Shapiro-Wilk and Kolmogorov-Smirnov tests. Descriptive statistics of numerical variables were presented as mean ± standard deviation and median (minimum - maximum). Mann-Whitney U and Kruskal-Wallis tests were used to compare numerical variables, and chi-square test was used to compare categorical variables. A p value <0.05 was considered statistically significant.

## Results

A total of 314 eyes of 255 patients who underwent PK for reasons other than MK between 2002 and 2019 in our clinic were evaluated. Of these, 36 eyes of 36 patients (11.5%) developed postoperative MK, 15 (41.7%) of which were in the early postoperative period. The mean age of patients with post-PK MK was 57.9±20 years (median 63.5 years, 10-85 years) and 58.3% were male. The mean ages of patients who developed MK in the early and late postoperative periods were 49.7±21.9 years (median 41 years, 10-82 years) and 63.9±16.6 years (median 68 years, 35-85 years), respectively (p=0.06). The proportion of males was similar in both groups (60% vs. 57.1%).

Twenty MK cases were bacterial (55.5%), 15 were viral (41.7%), and 1 was fungal (2.8%). Four patients had polymicrobial MK (2 gram-positive strains in 3 patients; gram-positive and gram-negative strains in 1 patient). None of the patients had a combination of bacterial/viral, bacterial/fungal, or viral/fungal keratitis.

The median time of MK development after PK was 10.5 months (1-39 months) for bacterial cases and 19 months (1-127 months) for viral cases; the difference was not statistically significant (p=0.06, Table 1). Among cases of viral keratitis, 73.3% occurred in the late postoperative period. When cases of bacterial MK were analyzed according to time to development and causative agents, the median time was 5 months (1-36 months) for gram-positive bacteria and 23 months (9-39 months) for gram-negative bacteria. Gram-positive bacteria were a significantly earlier MK agent compared to gram-negative bacteria (p=0.037).

Cultures were positive in all cases of bacterial and fungal MK except for 1 patient whose direct smear revealed gram-positive diplococci. The bacterial and fungal agents of MK isolated in culture are summarized in Table 2 according to their prevalence in the early and late postoperative periods. According to culture results, gram-positive bacteria were the most frequent, with the most common of these being coagulase-negative Staphylococcus species (33.3%). Of the gram-negative bacteria, Pseudomonas and Serratia were equally frequent (42.9%) (Table 2).

The antibiogram results of 14 gram-positive and 6 gram-negative bacteria were evaluated. Other than 1 strain that was not analyzed, all gram-positive bacteria were sensitive to vancomycin (Table 3). All gram-negative bacterial strains were sensitive to ciprofloxacin, and other than 1 strain that was not analyzed, all were sensitive to amikacin, ceftazidime, and gentamicin (Table 4).

In the only case of early MK (postoperative 1 month) with fungal growth in culture, Candida was detected and the patient had systemic candidiasis. Antibiogram showed the strain was sensitive to fluconazole and voriconazole.

The prevalence of diabetes mellitus (DM) was similar among patients with and without MK after PK (25% and 23.5%, respectively) (p=0.77).

Among patients who developed MK after PK, the most common indication for primary PK was pseudophakic bullous keratopathy (38.9%). Fifty percent (n=18) of patients who developed MK used antiglaucomatous medication, but the difference was not statistically significant when compared to PK patients who did not develop MK (38.2%) (p=0.10). In terms of the distribution of antiglaucomatous medications used by the patients, 10 (47.6%) were beta-blocker + alpha-2 agonist fixed combination, 8 (38%) were beta-blocker, 1 (4.8%) was alpha-2 agonist, 1 (4.8%) was carbonic anhydrase inhibitor, and 1 (4.8%) was carbonic anhydrase inhibitor + beta blocker fixed combination.

## Discussion

The prevalence of MK after PK varies between 1.8%^2^ and 12.1%^5^ in the literature, and was 11.5% in our study.

The median time from PK to development of bacterial MK was 10.5 months in our study, whereas this time varies between 9 and 17 months in other studies.^2,17,19^ Chen et al.^20^ observed in their study that most bacterial and fungal keratitis developed in the early postoperative period, while Sun et al.^10^ reported that more than half of MK cases occurred in the late postoperative period. In studies on viral keratitis, Remeijer et al.^21^ determined that most cases developed in the early postoperative period, whereas Rezende et al.^14^ found that keratitis often occurred in the last postoperative period. However, there are no studies in the literature comparing the post-PK timing of bacterial and viral MK or gram-positive and gram-negative bacterial MK development. In our study, the median times of bacterial and viral MK were 10.5 months (1-39 months), and 19 months (1-127 months) and the difference was not statistically significant (p=0.06). Gram-positive bacteria were more common in the early postoperative period, whereas gram-negative bacteria were more common in the late postoperative period, and this difference was statistically significant (p=0.037).

The most common causes of MK after PK are gram-positive bacteria found in the ocular surface flora.^3,10,19,22^ Although Staphylococcus aureus was reported as the most common gram-positive strain in several studies,^3,10,19^ we found that coagulase-negative Staphylococcus species were the most common, similar to the study by Davila and Mian^22^ (Table 2). The most common gram-negative bacterial causes of MK were Pseudomonas in some studies^10,22^ and Moraxella in others^2,6,19^, whereas Pseudomonas and Serratia were equally common in our study (Table 2).

Antibiotic susceptibility analysis of the MK agents showed that the gram-positive agents were sensitive to vancomycin and the gram-negative agents were sensitive to ciprofloxacin, amikacin, ceftazidime, and gentamicin (Tables 3 and 4). Given these results, combinations of vancomycin and the other 4 antibiotics can be used as empirical treatment for patients with suspected bacterial keratitis, due to their effectiveness against gram-positive and -negative bacteria.

Studies have demonstrated that fungal eye infections after keratoplasty may be caused by contaminated donor corneal tissue.^23,24^ The development of Candida keratitis at postoperative 1 month in our patient could also have been considered related to contamination of the donor cornea, but culture of the donor corneal rim was negative and the patient had systemic candidiasis.

Vajpayee et al.^25^ reported that DM was present in only 4 (8%) of the patients with MK after PK, whereas Saini et al.^26^ found that DM was associated with higher risk of MK in their study. In our patient group, 9 individuals (25%) had DM, and when all PK patients were evaluated, no significant difference in the prevalence of DM was detected between those with and without MK (p=0.77).

In glaucoma patients, the prevalence of common ocular surface diseases associated with preservatives or the active molecule increases with the number of antiglaucomatous drugs used.^27^ The ocular surface is an important factor in the development of MK.^9^ In this context, it can be argued that the use of antiglaucomatous agents may have an indirect effect in MK development. However, in our study there was no statistically significant difference between PK patients with and without MK in terms of antiglaucomatous drug use (p=0.10).

## Conclusion

In conclusion, considering that the majority (58.3%) of MK cases develop after more than 1 year, longer postoperative follow-up becomes more important for PK patients. When managing keratitis in patients with negative cultures, it should be kept in mind that gram-positive bacteria found in the ocular surface flora are more frequently the cause in the early postoperative period, while gram-negative bacteria and viral agents are more common in the late postoperative period.

## Figures and Tables

**Table 1:**
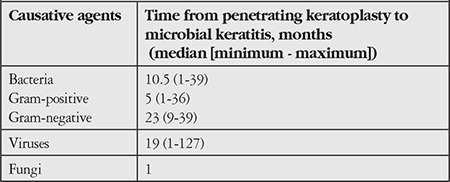
Time from penetrating keratoplasty to development of microbial keratitis

**Table 2:**
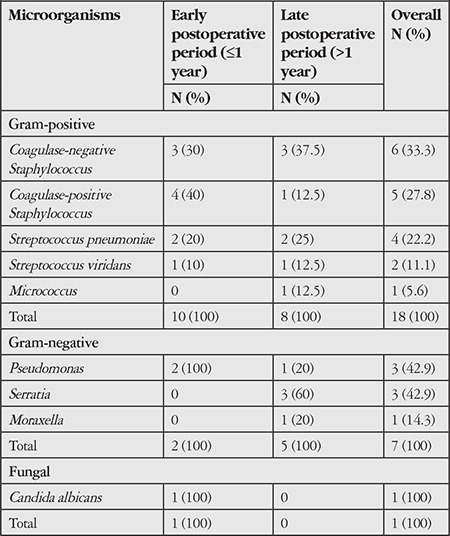
Distribution of microorganisms isolated in culture

**Table 3:**
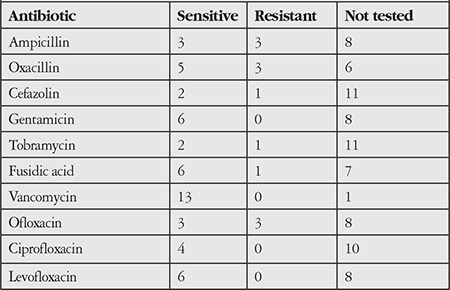
Antibiotic susceptibility of gram-positive causative agents

**Table 4:**
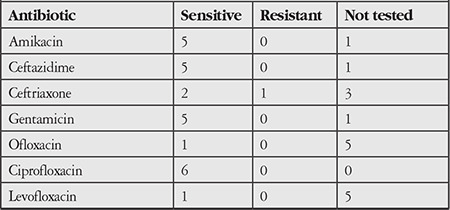
Antibiotic susceptibility of gram-negative causative agents
